# GO nanoparticles mitigate the negative effects of salt and alkalinity stress by enhancing gas exchange and photosynthetic efficiency of strawberry plants

**DOI:** 10.1038/s41598-023-35725-0

**Published:** 2023-05-25

**Authors:** Mohammad Reza Malekzadeh, Hamid Reza Roosta, Hazem M. Kalaji

**Affiliations:** 1 Department of Horticultural Sciences, Faculty of Agriculture, Vali-e-Asr University of Rafsanjan, Kerman, 7718817111 Iran; 2 Department of Horticultural Sciences, Faculty of Agriculture and Natural Resources, Arak University, Arak, 38156-8-8349 Iran; 3 Department of Plant Physiology, Institute of Biology, Warsaw University of Life Science, 159 Nowoursynowska St., 02-776 Warsaw, Poland; 4 Institute of Technology and Life Sciences—National Research Institute, Falenty, Al. Hrabska 3, 05-090 Raszyn, Poland

**Keywords:** Biological fluorescence, Plant stress responses, Photosystem I, Photosystem II

## Abstract

Considering the potential use of nanomaterials, particularly carbon-based nanostructures, in agriculture, we conducted a study to investigate the effect of graphene oxide (GO) on strawberry plants under salinity and alkalinity stress conditions. We used GO concentrations of 0, 2.5, 5, 10, and 50 mg/L, and applied stress treatments at three levels: without stress, salinity (80 mM NaCl), and alkalinity (40 mM NaHCO_3_). Our results indicate that both salinity and alkalinity stress negatively impacted the gas exchange parameters of the strawberry plants. However, the application of GO significantly improved these parameters. Specifically, GO increased PI, F_v_, F_m_, and RE_0_/RC parameters, as well as chlorophyll and carotenoid contents in the plants. Moreover, the use of GO significantly increased the early yield and dry weight of leaves and roots. Therefore, it can be concluded that the application of GO can enhance the photosynthetic performance of strawberry plants, and improve their resistance to stress conditions.

## Introduction

Nanomaterials are molecules with a size ranging from 1 to 100 nm^1^, and graphene is an atomically thick, two-dimensional crystal made up of single-layer carbon atoms^2^. Graphene oxide (GO) is a monolayer of graphene with a thickness of 2–3 nm, slightly thicker than monolayer graphene due to the presence of functional groups containing oxygen, which changes the surface of the GO sheet and makes it thicker^3^. GO-based materials have unique physical and chemical properties, attracting attention in various fields^4^. In an experiment, a high concentration of GO (500–2000 mg/L) significantly inhibited the growth of tomato, red spinach, cabbage, and lettuce, indicating that the toxicity of Graphene-based materials for plants is related to the concentration and plant species^5^. However, GO has also been shown to stimulate the growth of coriander and garlic plants^6^, highlighting its potential as a regulatory tool to improve the growth and development of plants.

Plants face many environmental stresses, and high salinity and alkalinity of soil and water are one of the most significant and widespread abiotic stresses for agriculture worldwide, causing severe damage to crop growth and development^7^. High salinity and alkalinity harm plant growth by preventing leaf expansion, stomata closure, and development, and affecting physiological processes such as photosynthesis, transpiration, respiration, enzyme activity, nutrient balance, and pH of the culture medium^8^. Salinity stress produces reactive oxygen species (ROS) that are toxic to the cell and can damage carbohydrates, proteins, and lipids^9^. It also causes an ionic imbalance in the cell, leading to the toxicity of ions and osmotic stress on plants^10^. The accumulation of salt in the mesophyll cells results in the absorption of carbon dioxide and an increase in CO_**2**_ concentration inside the leaf, leading to a decrease in stomatal conductance^11^.

GO applications have beneficial effects on the growth and development of plants, such as an increase in photosynthetic pigments, proline, soluble carbohydrates, enzyme activity, phenol, flavonoids, glutathione, and ascorbic acid^12^, which have a positive role in stress conditions in plants^13^. The survival of plants under stress conditions depends on the generation and transmission of signals, the ability of plants to perceive the stimulus, and the triggering of biochemical changes that regulate metabolism accordingly and control stress-adaptive responses in plants^14^. Graphene oxide treatment could reduce the adverse effects of salinity by improving the biochemical and physiological responses of Sultana grapes^15^.

Analyzing the effects of GO application on plant growth at the morphological, biochemical, photosynthetic, and molecular levels is necessary to optimize its applications in agriculture. The use of GO can have positive effects on plants under stress conditions, particularly salinity and alkalinity of water and soil. This study aims to investigate the effect of different concentrations of GO on the growth and development and photosynthetic system of the strawberry plant. The study focuses on using GOs to investigate the performance of the photosynthetic apparatus and the growth and development of strawberry plants under salinity and alkalinity stress conditions. The hypothesis is that GOs can improve plant growth by enhancing the performance of the photosynthetic apparatus and the electron transport chain. The findings can help set guidelines for applying GO in plants and provide new insights into how GO affects plants.

## Methods

### Plant material and growth conditions

In 2022, the experiment was conducted in the research greenhouse at Vali-e-Asr University of Rafsanjan- Iran (Latitude: 30° 21′ 17.6004'' N, Longitude: 56° 0′ 9.738'' E, Elevation: 1545.924). Bare root plants of strawberry (*Fragaria* × *ananassa* Duch, cv. Paros) were obtained from a nursery in Karaj, Iran. Plants were planted in a pot of 4 L containing cocopeat and perlite (70:30 ratio). Each treatment included three pots, and each pot was an experimental unit in which two plants were planted. Plants were cultivated in the greenhouse with a temperature of 25/15 ± 2 °C (day/night), a photoperiod of 11.13 h (light/dark), and relative humidity of 50 ± 10%. During this period, plants were fertigated with the Morgan nutrient solution^16^ (Electrical Conductivity (EC): 1.4 ds m^−1^ and pH: 6.5). The nutrient solution was prepared in a 200 L tank and supplied to the plant with a pump and through a dripper. Fertigation was done three times a day, and 300 ml of the nutrient solution was given to the plants every day. Plants were treated with three stress levels, including without stress, alkalinity (40 mM NaHCO_3_), and salinity (80 mM NaCl). The alkalinity and salinity treatments were applied twenty days after planting. 100 ml of NaHCO_3_ and NaCl were added to each pot every 3 days to maintain constant stress for the plants. Stresses continued until the end of the experiment and the completion of data collection. The drainage of the bed was measured every day, and with the increase of EC, Leaching was done so that the EC was constant during the experiment at the expected level.

### GO treatment

A modified Hammers method^17,18^ was used to make GO (GO) from graphite powder. First, the mixture containing 2.0 g of graphite powder and 46 ml of H_2_SO_4_ (98%) was placed in an ice water bath. Then, 6.0 g of KMnO_4_ was added to the mixture suspension under stirring to obtain a green mixture. Then, it was transferred to an oil bath with a temperature of 40 degrees Celsius and stirred until a brown paste was obtained. Then 90 ml of water was added, and the resulting mixture was poured into deionized water under stirring. Next, 35% H_2_O_2_ was added to the reaction mixtures to remove residual KMnO_4_ until a yellow color was obtained, confirming the conversion of graphite oxide from graphite. The yellow products were washed with HCl (1 M) and deionized water to remove metal ions. Finally, the mixtures were dried at room temperature.

GO forms a dispersion in water. Thus, GO was dispersed in water using an ultrasonic bath and different concentrations were prepared. Plants were treated with five GO concentrations (0, 2.5, 5, 10, and 50 mg/L). The GO treatments were applied twenty days after planting. GO treatment was applied as a foliar application once a week.

### Characterization of GO

**Figure 1:**
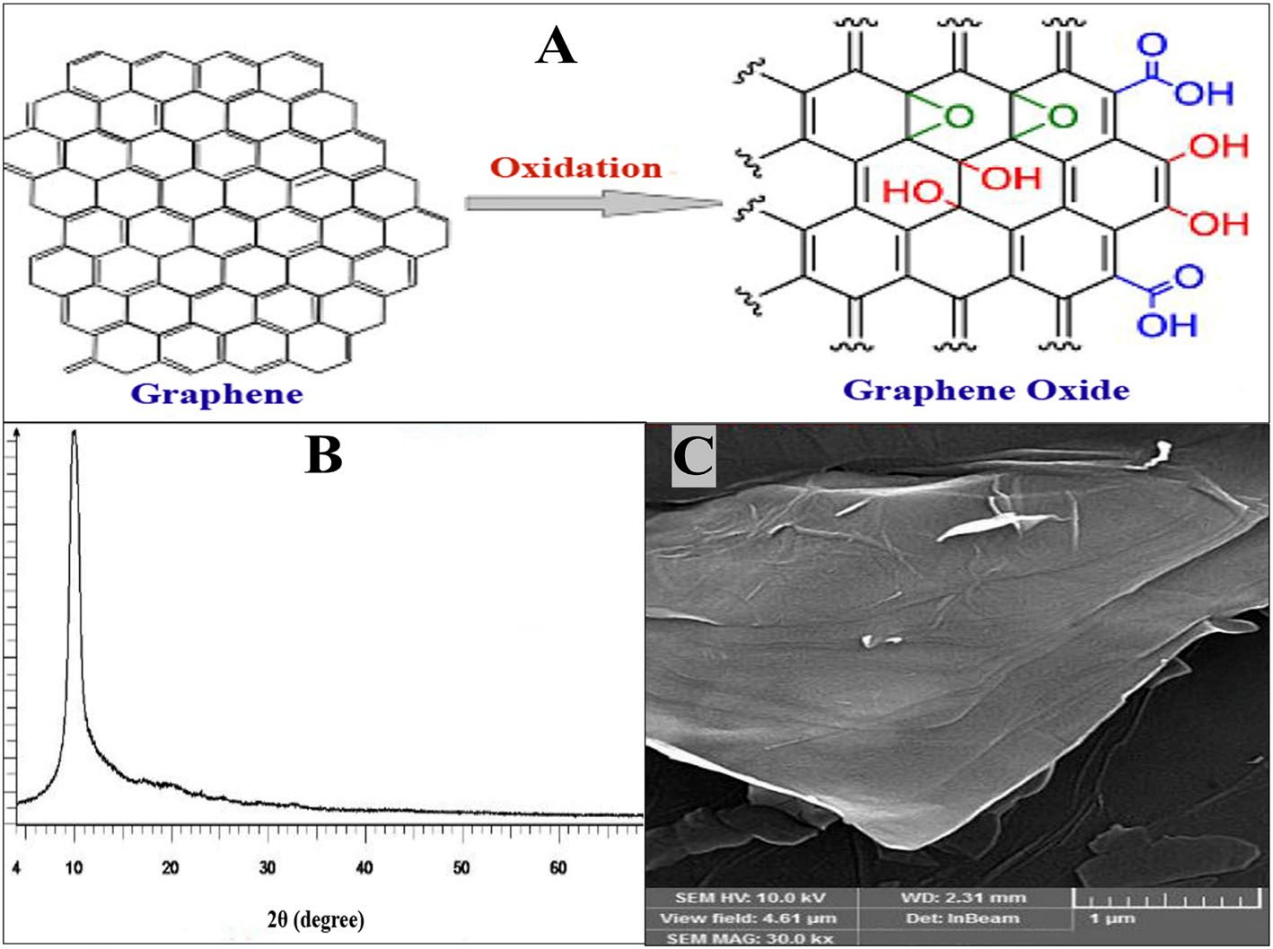
The chemical structure of a small part of a graphene molecule and its oxidation to GO (**A**). The XRD spectra of the synthesized GO (**B**). The morphology of GO nanomaterial by SEM (**C**).

The schematic structure of a small part of the graphene molecule and its oxidation to GO is shown (Fig. 1A). The XRD spectra of the synthesized nanoparticles are shown in Fig. 1B. The peak observed at 2ϴ (equals 11°) confirms the synthesis of GO. The morphology of GO nanomaterial was determined by SEM. Figure 1C shows the SEM image of graphene nanoparticles. This image reveals that the graphene oxide plates were well-synthesized.

### Evaluation of chlorophyll fluorescence

We used a portable photosynthetic efficiency analyzer (PEA, Hansatech Inc. Co., UK) sixty days after planting to measure and calculate chlorophyll fluorescence parameters. For this purpose, fully mature leaves of each pot were adapted to a dark time for 15 min by fixing special tags on each leaf before measurement. Then the sensor cup was mounted on the leaf for calculation. The chlorophyll *a* fluorescence transient was caused by a saturating photon flux density of 3500 μmol (photon) m^−2^.s^−1^ given by three light-emitting diodes (peak 650 nm) to produce fluorescence curves ranging from F_0_ to F_m_ (F_t_, fluorescence at time t after the onset of actinic illumination; F_0_ = F_30μs_, minimum fluorescence intensity; F_j_ = F_2ms_, fluorescence intensity at the J-step; F_i_ = F_30ms_, fluorescence intensity at the I-step; F_p_ = F_m_, Maximum fluorescence intensity at peak P of OJIP) for all treatments^19^. The PSII parameters obtained from the OJIP transient were evaluated based on the Strasser et al.^10^ methods. Parameters for chlorophyll fluorescence are listed in Table S1.

### Leaf gas exchange

Plant gas exchange parameters include net CO_2_ assimilation rate (*A*, μmol (CO_2_) m^−2^ s^−1^), water-use efficiency (*WUE* (*A*/*E*), μmol CO_2_ mol H_2_O^−1^), stomatal conductance (*g*_*s*_, mol H_2_O m^−2^.s^−1^), transpiration (*E*, mmol H_2_O m^−2^.s^−1^), Sub-stomatal CO_2_ concentration (*C*_*i*_, μmol CO_2_ mol^-1^) and, instantaneous carboxylation efficiency (*A/C*_*i*_) were measured using a portable photosynthesis system (ADC BioScientific Ltd, Hoddesdon, UK) 60 days after planting. Measurements were made 30 days after applying the treatments and when the leaves were mature. Around 9:00 AM and 12:00 AM, measurements were performed on completely expanded leaves^19^.

### Photosynthetic pigments, carotenoids, SPAD value, and leaf relative water content (LRWC)

To determine the chlorophyll (total chlorophyll, chlorophyll *a*, and chlorophyll *b*) and carotenoid contents, three fully extended leaves from each pot were collected. The extract was prepared from fresh leaves (0.25 g) by grinding in a cold mortar together with 10 ml of 80% aqueous acetone. After filtering, the absorbance of centrifuged extracts was measured at 470, 646, and 663 nm using spectrophotometer-U-2000, Hitachi Instruments, Tokyo, Japan^20^. The chlorophyll index in young leaves was recorded with a SPAD-502 Chlorophyll Meter (Minolta Camera Co. Ltd., Osaka, Japan). Three leaves were selected from each pot, and measurements were made. Fresh leaves were used to determine the LRWC. One leaf from the fully expanded leaf was cut from each plant. Leaf disks (5 mm diameter) were obtained from the leaves. To determine the fresh mass (FM), leaf discs were prepared and weighed. Then, it floated on distilled water in a petri dish and incubated at normal room temperature. After four hours, the adhering water of the discs was blotted and then weighed to determine turgor mass (TM). The samples were dried at 70 °C for 24 h, and the dry mass (DM) was obtained^21^. Relative water content was calculated using the following equation:

$$ \left( {{\text{LRWC}}\,{\text{in}}\,\% } \right)\, = \,\left[ {\left( {{\text{FM }} - {\text{ DM}}} \right)/\left( {{\text{TW }} - {\text{ DM}}} \right)} \right]\, \times \,{1}00. $$

FW: fresh mass, DW: dry mass, TW: turgor mass.

### Vegetative and reproductive characteristics and total soluble solids of fruit

At the end of the experiment, plants were harvested for measurement. Their branches, roots, and crowns were separated. To measure dry mass, the samples were placed in an oven at 70 °C for 72 h, and then the dry mass of the samples was recorded. Fruit number and early yield are measured during the growth period of plants. The total soluble solids (° Brix) of fruits were measured with a refractometer (PAL-1, Atago Co., Ltd; Japan)^21^.

### Experimental design and data analysis

The experiment was factorial, consisting of a randomized, complete design with two factors in three replicates and two single plants per pot. All data were analyzed using SAS software version 9.4 (SAS Institute, Cary, NC, USA. https://www.sas.com/en_us/home.Html). When variance analysis (ANOVA) indicated significant treatment effects, significant mean variations (P < 0.05) were calculated using the LSD Multiple Range Test. Biophysical parameters were determined with “PEA Plus” software version 1.12 (http://www.hansatech-instruments.com). Pearson's correlation coefficient was applied to determine the relationships among the parameters studied. Principal component analysis was performed using XLSTAT software version R2015b (https://www.xlstat.com/). A correlation plot was drawn with Origin Pro software version 2022 (https://www.originlab.com/2022). The biplots were made using Excel software version 2016 (https://www.microsoft.com).

### Statement of compliance

The authors confirm that all the experimental research and field studies on strawberry plants, including the collection of plant material, complied with relevant institutional, national, and international guidelines and legislation. Also, obtained licenses for the preparation of Bare root plants of strawberry.

## Results

### Leaf gas exchange analyses

**Figure 2:**
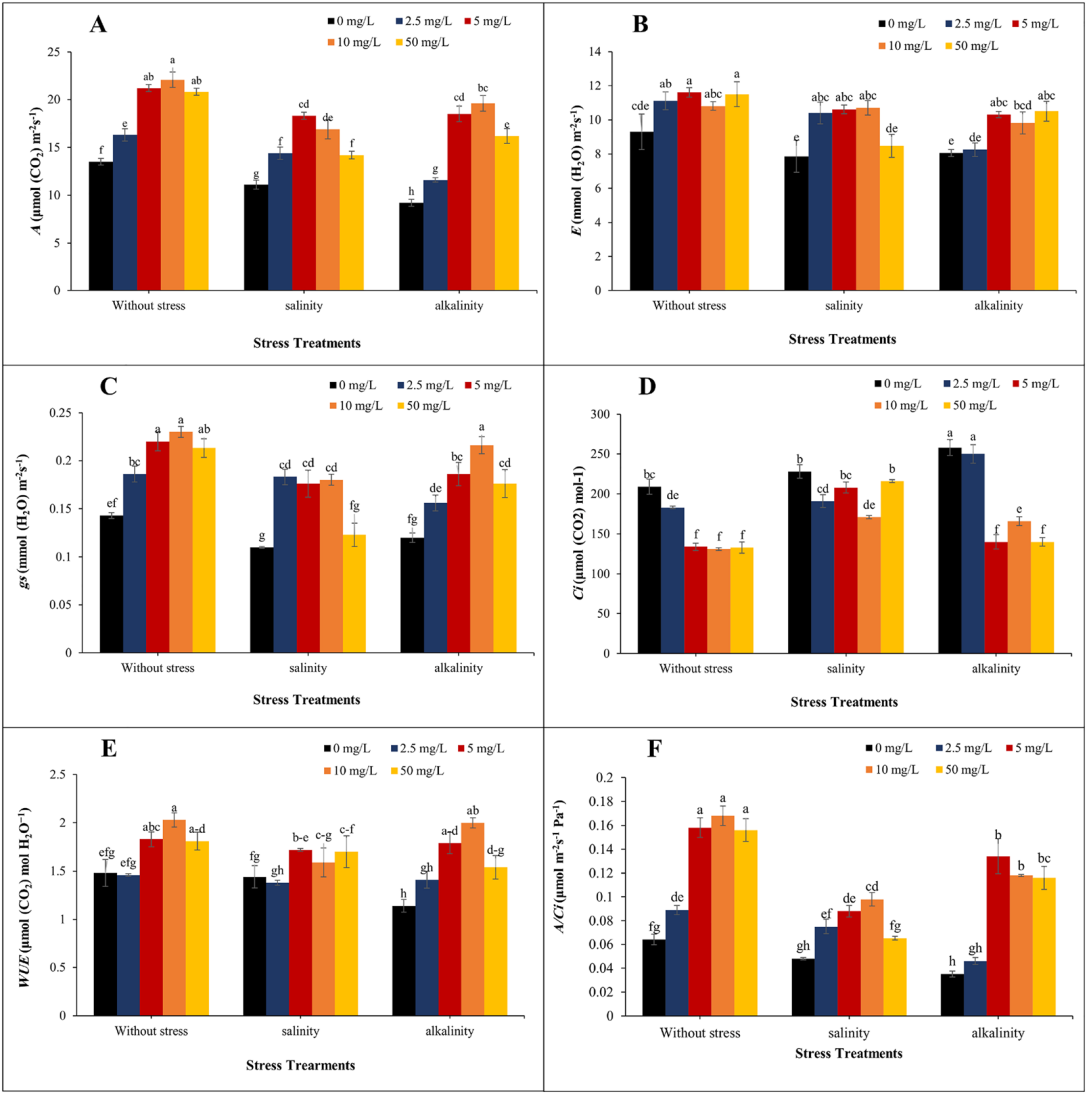
Leaf Gas Exchange parameters of different GO concentrations on strawberry cv. Sabrina under salinity and alkalinity stress conditions. (**A**) CO_2_ Assimilation Rate (*A*); (**B**) Transpiration rate (*E*); (**C**) Stomatal conductance (*g*_*s*_); (**D**) sub-stomatal CO_2_ concentration (*C*_*i*_); (**E**) Water-use efficiency (*WUE*); (**F**) Instantaneous carboxylation efficiency (*A/C*_*i*_). Means followed by the same letter for a parameter, are not significantly different according to the LSD (p ≤ 0.05). Vertical bars indicate the standard errors of three replicates.

Our results showed that leaf gas exchange parameters were affected by different concentrations of GO and stress conditions. Salinity and alkalinity stress conditions decreased CO_2_ assimilation rate (*A*), transpiration rate (*E*), stomatal conductance (*g*_*s*_), water-use efficiency (*WUE*), instantaneous carboxylation efficiency (*A/C*_*i*_), and increased sub-stomatal CO_2_ concentration (*C*_*i*_). Concentrations of 5 and 10 mg/L of GO had the most significant effect on the CO_2_ assimilation rate under without stress and alkalinity treatment, and had no significant difference with the concentration of 5 mg/L. In salinity treatment, the concentration of 5 mg/L GO had the greatest effect on increasing the CO_2_ assimilation rate, which was not significantly different from the concentration of 10 mg/L (Fig. 2A). In salinity stress, the application of 2.5, 5, and 10 mg/L, and in alkalinity stress, the application of 5, 10, and 50 mg/L had the greatest effect on transpiration rate, and in without stress condition, different concentrations of GO did not show significant differences from each other (Fig. 2B). In the without stress treatment, the concentration of 10 mg/L of GO had the greatest effect on stomatal conductance, and in the salinity treatment, the concentrations of 2.5, 5 and 10 mg/L had a significant effect on the increase of stomatal conductance, without any significant differences with each other (Fig. 2C). The stress condition increased the Sub-stomatal CO_2_ concentration, and the application of GO reduced it. The treatments of 5, 10, and 50 mg/L of GO had the greatest effect on reducing Sub-stomatal CO_2_ concentration in the without stress treatment and alkalinity stress. In the salinity stress conditions, the concentration of 10 mg/L of GO had the greatest effect on reducing Sub-stomatal CO_2_ concentration (Fig. 2D). The highest level of water-use efficiency was observed by applying 10 mg/L in without stress treatment. In salinity stress, the highest water-use efficiency was obtained at concentrations of 5 mg/L, which were not significantly different from concentrations of 10 and 50 mg/L. In alkalinity stress conditions, the application of 10 mg/L of GO had the greatest effect on increasing the water-use efficiency (Fig. 2E). In the without stress treatment, concentrations of 5, 10, and 50 mg/L GO had the greatest effect on A/Ci, which were not significantly different from each other. Although the stress conditions reduced A/C_i_, the application of 10 mg/L in salinity stress conditions and 5 mg/L of GO in alkaline stress, reduced the effects of stress more than other concentrations of GO (Fig. 2F).

### Prompt chlorophyll a fluorescence

**Figure 3:**
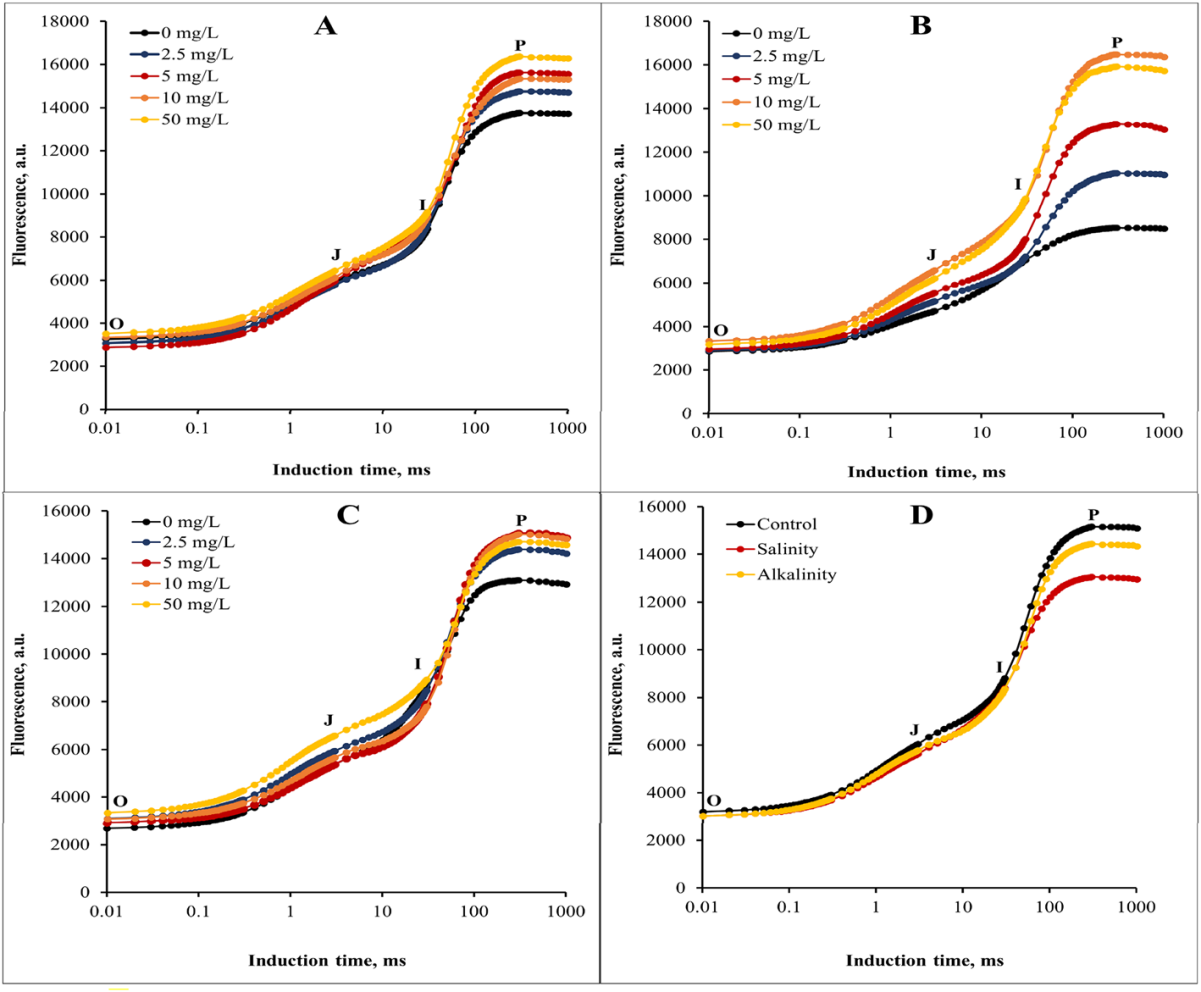
Induction curves of chlorophyll *a* fluorescence of strawberry cv. Sabrina. (**A**) the effect of different GO concentrations (0, 2.5, 5, 10, and 50 mg/L) under without stress conditions; (**B**) the effect of different GO concentrations (0, 2.5, 5, 10, and 50 mg/L) under salinity stress; (**C**) the effect of different GO concentrations (0, 2.5, 5, 10, and 50 mg/L) under alkalinity stress; (**D**) Independent effects of salinity and alkalinity stress under different GO concentrations on chlorophyll fluorescence induction curves.

In without stress conditions, the concentration of 50 mg/L GO had the greatest effect on the increase of fluorescent transients (Fig. 3A). In salinity stress, the application of 10 and 50 mg/L GO caused a significant increase in J, I, and P points (Fig. 3B). In the alkalinity stress, the application of 5 and 10 mg/L GO concentrations increased the P point. The 50 mg/L GO concentration increased the J point compared to the other concentrations of GO (Fig. 3C). Stress conditions significantly affected the fluorescent transients compared to without stress conditions. Salinity stress had a greater effect on reducing the fluorescence curve at the P point than alkalinity stress (Fig. 3D).

### Chlorophyll fluorescent transients and calculated curves

**Figure 4:**
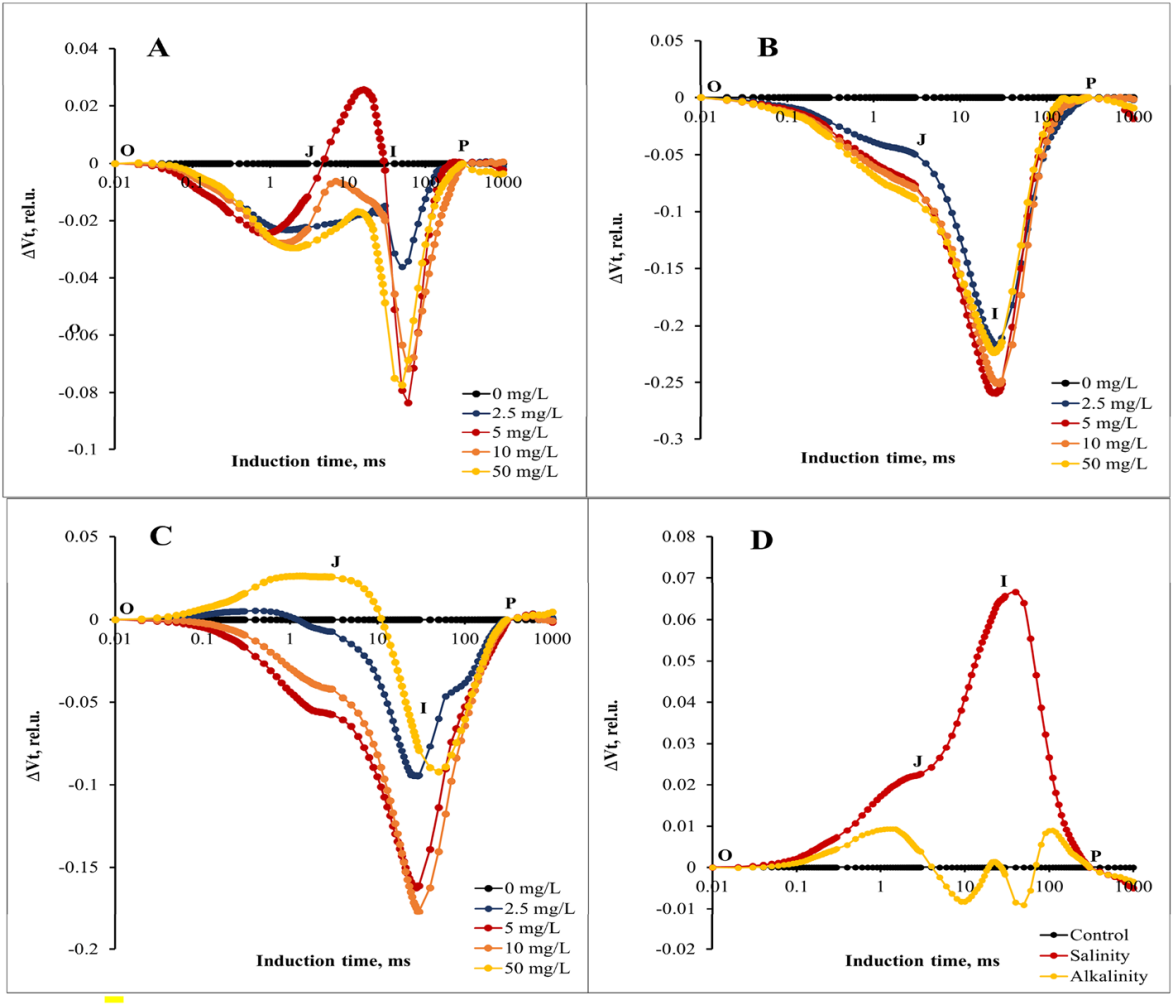
Differential curves of ΔV_t_ of chlorophyll *a* fluorescence of strawberry cv. Sabrina. (**A**) the effect of different GO concentrations (0, 2.5, 5, 10, and 50 mg/L) under without stress conditions; (**B**) the effect of different GO concentrations (0, 2.5, 5, 10, and 50 mg/L) under salinity stress; (**C**) the effect of different GO concentrations (0, 2.5, 5, 10, and 50 mg/L) under alkalinity stress; (**D**) Independent effects of salinity and alkalinity stress under different GO concentrations on Differential curves of ΔV_t_ of chlorophyll *a* fluorescence.

The relative variable fluorescence curve was created to explore the effects of different GO concentrations and stress interaction on transient dynamics, Vt = (F_t_ − F_O_)/ (F_M_ − F_O_)^10^. Next, the changes in the OJIP fluorescence were calculated by subtracting the values of the fluorescence (O–P) recorded in plants under stress from those recorded in without stress plants. According to our results, the variable fluorescence changes under the stress conditions (Fig. 4B,C) were different from the without stress condition (Fig. 4A), so the changes of the relative variable fluorescence curve under the influence of different concentrations of GO under stress conditions were more than the without stress treatment, and different concentrations of GO had different effects at different points of the curve. Relative variable fluorescence (ΔVt) changes were higher under salinity stress (especially in I point) than alkalinity stress condition (Fig. 4D).

**Figure 5:**
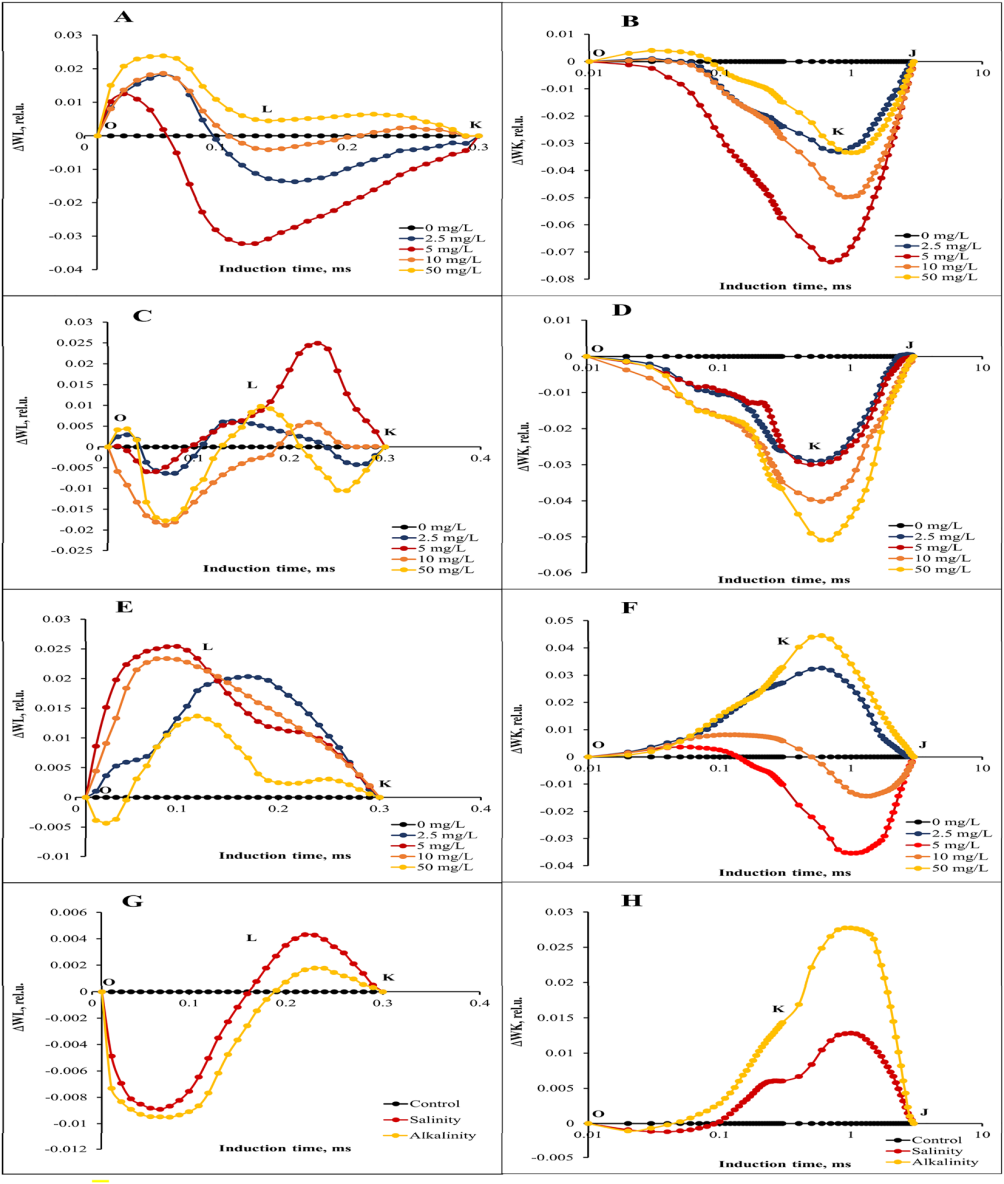
Differential curves of ΔW_L_ and ΔW_K_ of chlorophyll *a* fluorescence of strawberry cv. Sabrina. (**A**) and (**B**) the effect of different GO concentrations (0, 2.5, 5, 10, and 50 mg/L) under without stress conditions; (**C**) and (**D**) the effect of different GO concentrations (0, 2.5, 5, 10, and 50 mg/L) under salinity stress; (**E**) and (**F**) the effect of different GO concentrations (0, 2.5, 5, 10, and 50 mg/L) under alkalinity stress; (**G**) and (**H**) Independent effects of salinity and alkalinity stress under different GO concentrations on Differential curves of ΔW_K_ and ΔW_L_ of chlorophyll *a* fluorescence.

For a detailed evaluation of changes in GO concentrations under stress conditions in OJIP fluorescence kinetics, we provide differential curves for the L, K, H, and G bands that occur during the transient O to J points. The curves of these bands were calculated by subtracting the amount of normal fluorescence (between O-K, O-J, J-I, and I-P points, respectively) recorded in the without stress plants from that recorded in the plants under different GO concentrations and stress conditions. Our results showed that different concentrations of GO had different effects on L and K bands under stress and without stress conditions. In without stress conditions, the concentration of 5 mg/L of GO had the greatest effect on the reduction of L and K bands (Fig. 5A,B). In the conditions of salinity stress, the concentration of 10 mg/L of GO had the greatest effect on the reduction of L and K bands (Fig. 5C,D). In alkalinity stress conditions, concentrations of 5 and 50 mg/L had the greatest effect on the reduction of L and K bands, respectively. (Fig. 5E,F). The results showed that both salinity and alkalinity stress increased L and K bands (especially the K band) (Fig. 5G,H).

**Figure 6:**
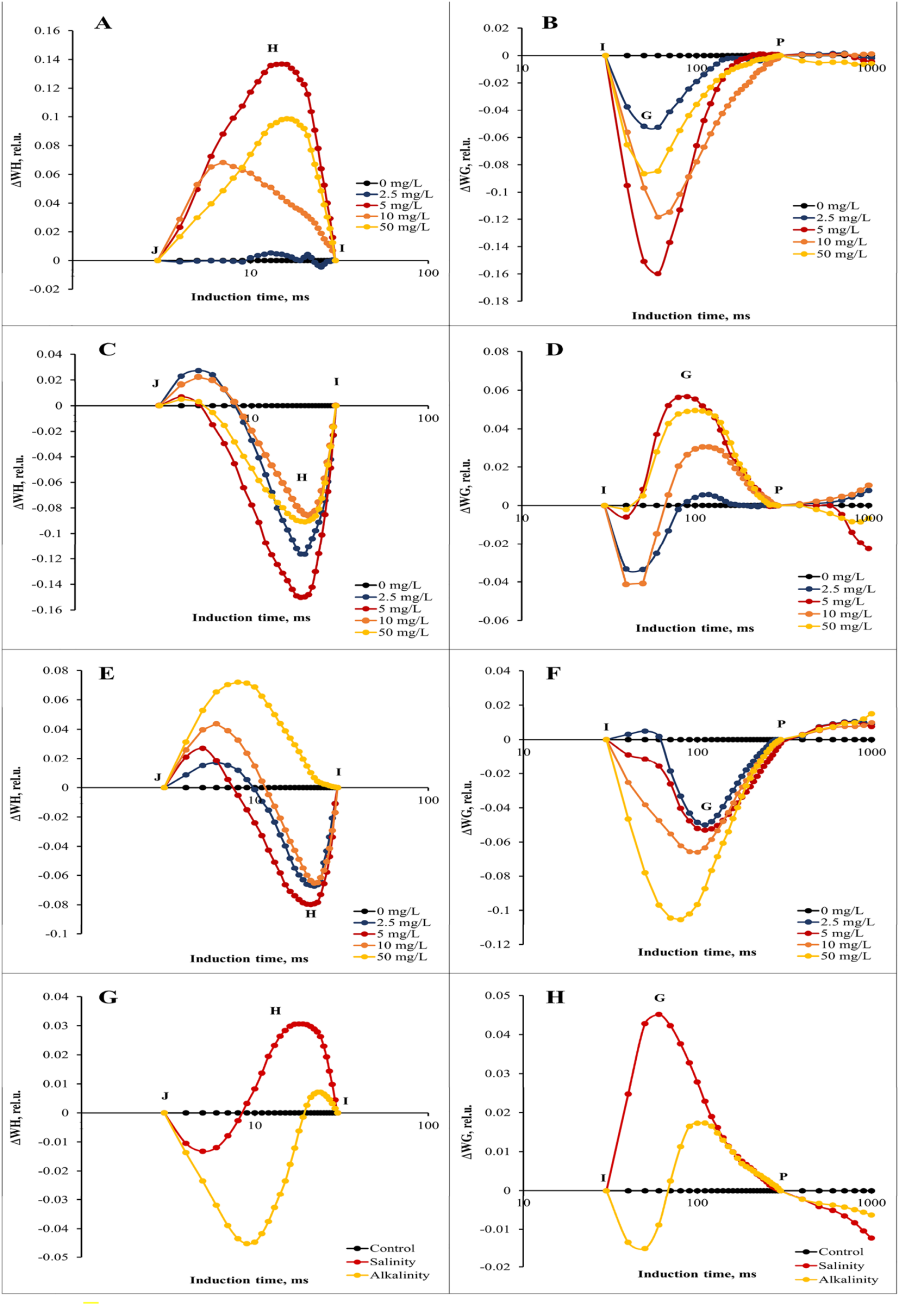
Differential curves of ΔW_H_ and ΔW_G_ of chlorophyll *a* fluorescence of strawberry cv. Sabrina. (**A**) and (**B**) the effect of different GO concentrations (0, 2.5, 5, 10, and 50 mg/L) under without stress conditions; (**C**) and (**D**) the effect of different GO concentrations (0, 2.5, 5, 10, and 50 mg/L) under salinity stress; (**E**) and (**F**) the effect of different GO concentrations (0, 2.5, 5, 10, and 50 mg/L) under alkalinity stress; (**G**) and (**H**) Independent effects of salinity and alkalinity stress under different GO concentrations on Differential curves of ΔW_H_ and ΔW_G_ of chlorophyll *a* fluorescence.

Concentrations of 5 and 2.5 mg/L GO had the greatest effect on increasing H and G bands, respectively (Fig. 6A,B). In salinity stress conditions, applying 5 and 10 mg/L of GO caused the greatest increase in H and G bands, respectively (Fig. 6C,D). In alkalinity stress, the concentration of 50 mg/L GO had the most effective in increasing the H band. The concentration of 2.5 mg/L GO increased the G band to a greater more than the other concentrations of GO (Fig. 6E,F). In general, salinity stress had a greater effect than alkalinity stress on H and G bands (Fig. 6G,H).

### JIP-test parameters, calculated from chlorophyll fluorescence transients

**Figure 7:**
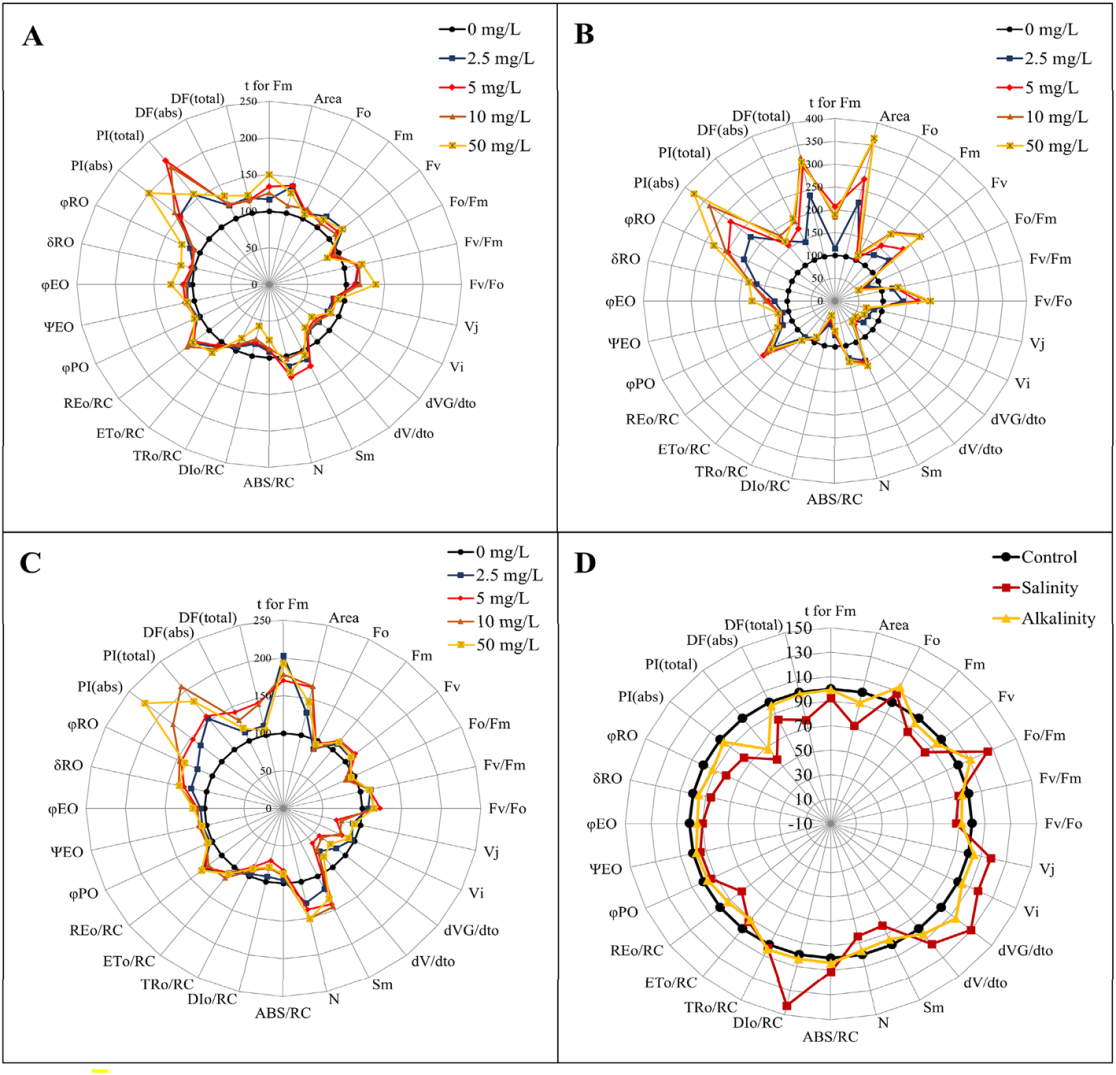
JIP-test parameters normalized on radar plots. (**A**) the effect of different GO concentrations (0, 2.5, 5, 10, and 50 mg/L) under without stress conditions; (**B**) the effect of different GO concentrations (0, 2.5, 5, 10, and 50 mg/L) under salinity stress; (**C**) the effect of different GO concentrations (0, 2.5, 5, 10, and 50 mg/L) under alkalinity stress; (**D**) Independent effects of salinity and alkalinity stress under different GO concentrations on JIP-test parameters.

OJIP transients have been translated into biophysical parameters: The basic parameters derived from the extracted data, normalized data, specific energy fluxes (per Q_A_ reduced PSII reaction center), quantum yield for primary photochemistry, slopes and integrals, and performance indexes^10^. The values of the measured parameters have been normalized to those of the without stress plants. On radar plots, the deviation of the activity pattern of plants under stress and different light spectra from without stress plants were shown (Fig. 7).

Our results showed that JIP-test parameters were affected by different concentrations of GO and stress conditions. Based on the obtained results, in without stress conditions, the concentration of 50 mg/L GO has a significant effect on increasing the parameters of PI_(abs)_, t for F_m_, and RE_0_/RC, and decreasing ABS/RC, DI_0_/RC, and F_0_/F_m_. The concentration of 5 mg/L of GO caused a significant increase in Pi_(total)_, S_m,_ and N (Fig. 7A). Under salinity stress conditions, the concentration of 50 mg/L GO caused a significant increase in Area and PI_ABS_. Concentrations of 10 and 50 mg/L of GO significantly increased F_V_ and F_m_ compared to other concentrations of GO (Fig. 7B). Under alkalinity stress, the concentration of 5 mg/L GO had a significant effect on decreasing DI_0_/RC parameter. The application of GO caused a significant increase in S_m_, N, and RE_0_/RC compared to the without stress treatment (Fig. 7C). The results showed that the application of GO had a positive effect on PSI and PSII and the stress conditions had a destructive effect on them. Salinity stress caused a significant increase in F_0_/F_m_, V_J_, V_i_, dVG/dt_0_, dv/dt_0_, and DI_0_/RC and decreased Area, F_v_, F_m_, RE_0_/RC, and quantum performance parameters compared to the without stress. Alkalinity stress decreased Area, F_m_, F_v_, PI_(total)_, ET_0_/RC, and RE_0_/RC and increased F_0_ and dVG/dt_0_ parameters (Fig. 7D).

### Photosynthetic pigments, carotenoids, SPAD values, and leaf relative water content (LRWC)

**Figure 8:**
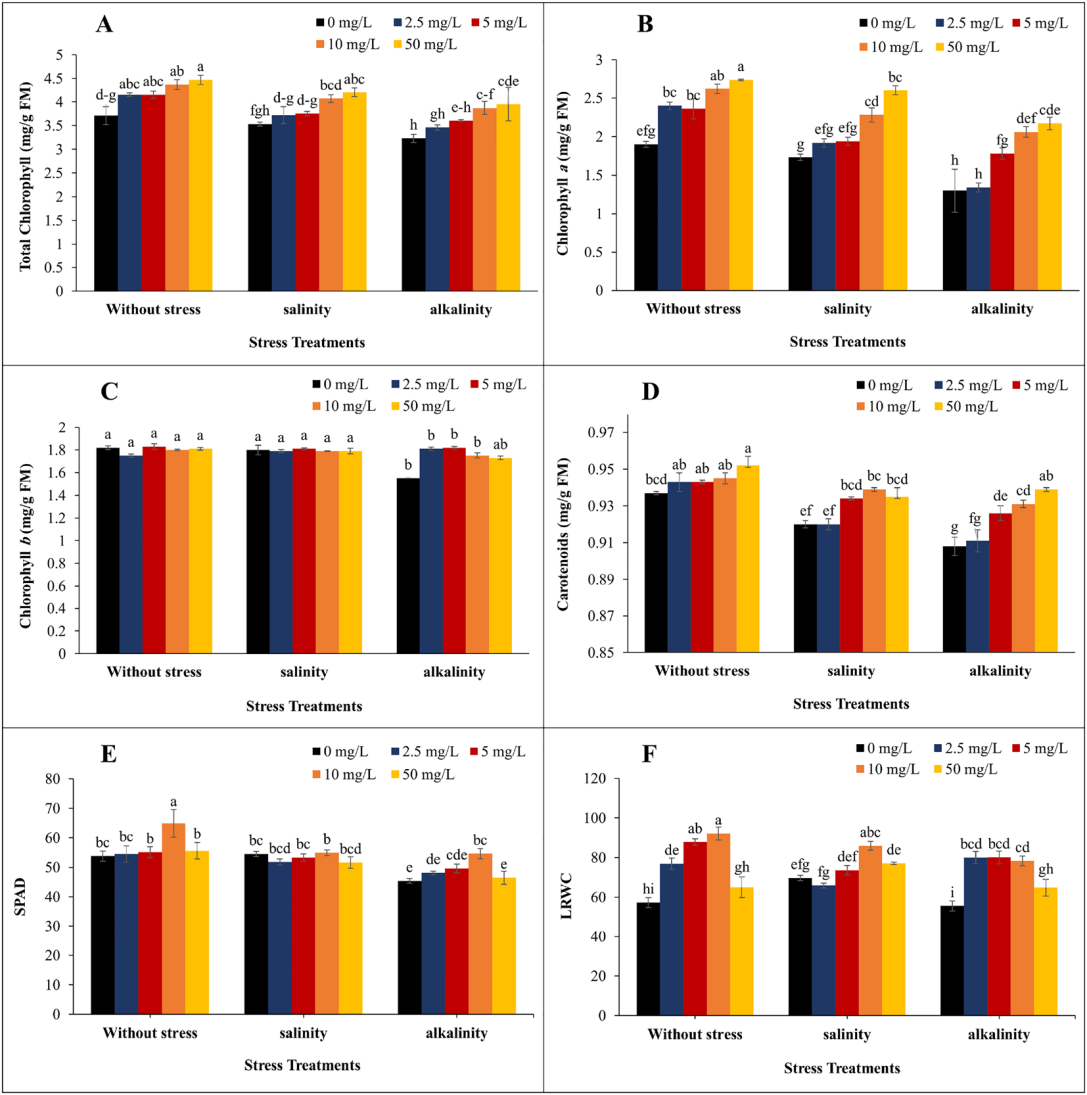
Changes in (**A**) total chlorophyll; (**B**) chlorophyll *a*; (**C**) chlorophyll *b*; (**D**) carotenoids; (**E**) SPAD value; (**F**) leaf relative water content (LRWC) under different GO concentrations and three stress levels in strawberries cv. Sabrina. Means followed by the same letter for a parameter, are not significantly different according to the LSD (p ≤ 0.05). Vertical bars indicate the standard errors of three replicates.

Stress conditions and the application of GO had a significant effect on chlorophyll, carotenoid, SPAD, and LRWC. The results showed that the highest amount of total chlorophyll and chlorophyll a was obtained in all stress conditions at a concentration of 50 mg/L of GO so that the amount of total chlorophyll in salinity and alkalinity stress increased by 18.9 and 22.2%, respectively, compared to the treatment without the use of GO. The highest increase in chlorophyll a was obtained in alkalinity conditions and the concentration of 50 mg/L of GO with 66.9% (Fig. 8A,B). Although the amount of chlorophyll b decreased under salinity and alkalinity stress, there was no significant difference between different concentrations of GO (Fig. 8C). Salinity and alkalinity stress reduced carotenoids, but with increasing the concentration of GO, the carotenoids increased. In the without stress and alkalinity treatment, the highest amount was obtained at a concentration of 50 mg/L of GO. In salinity stress, the highest amount was obtained at the concentration of 10 mg/L of GO, which was not significantly different from the concentrations of 5 and 50 mg/L of GO (Fig. 8D).

The highest increase in SPAD compared to the treatment without GO was recorded in the without stress treatment, and the concentration of 10 mg/L of GO with a 20.6% increase. In salinity stress, no significant difference was observed between GO concentrations, and in alkalinity stress, the highest increase in SPAD belonged to the treatment of 10 mg/L GO (20.2%) (Fig. 8E). The use of GO had a significant effect on LRWC. The highest amount was obtained in the without stress treatment with 10 mg/L of GO. Under salinity stress conditions, the highest increase in LRWC compared to the without stress treatment was obtained at a concentration of 10 mg/L of GO (23.2%). In the alkalinity stress, the highest LRWC was related to the concentrations of 2.5, 5, and 10 mg/L of GO, which did not significantly difference from each other (Fig. 8F).

### Vegetative growth and reproductive characteristics and total soluble solids of fruit

**Figure 9:**
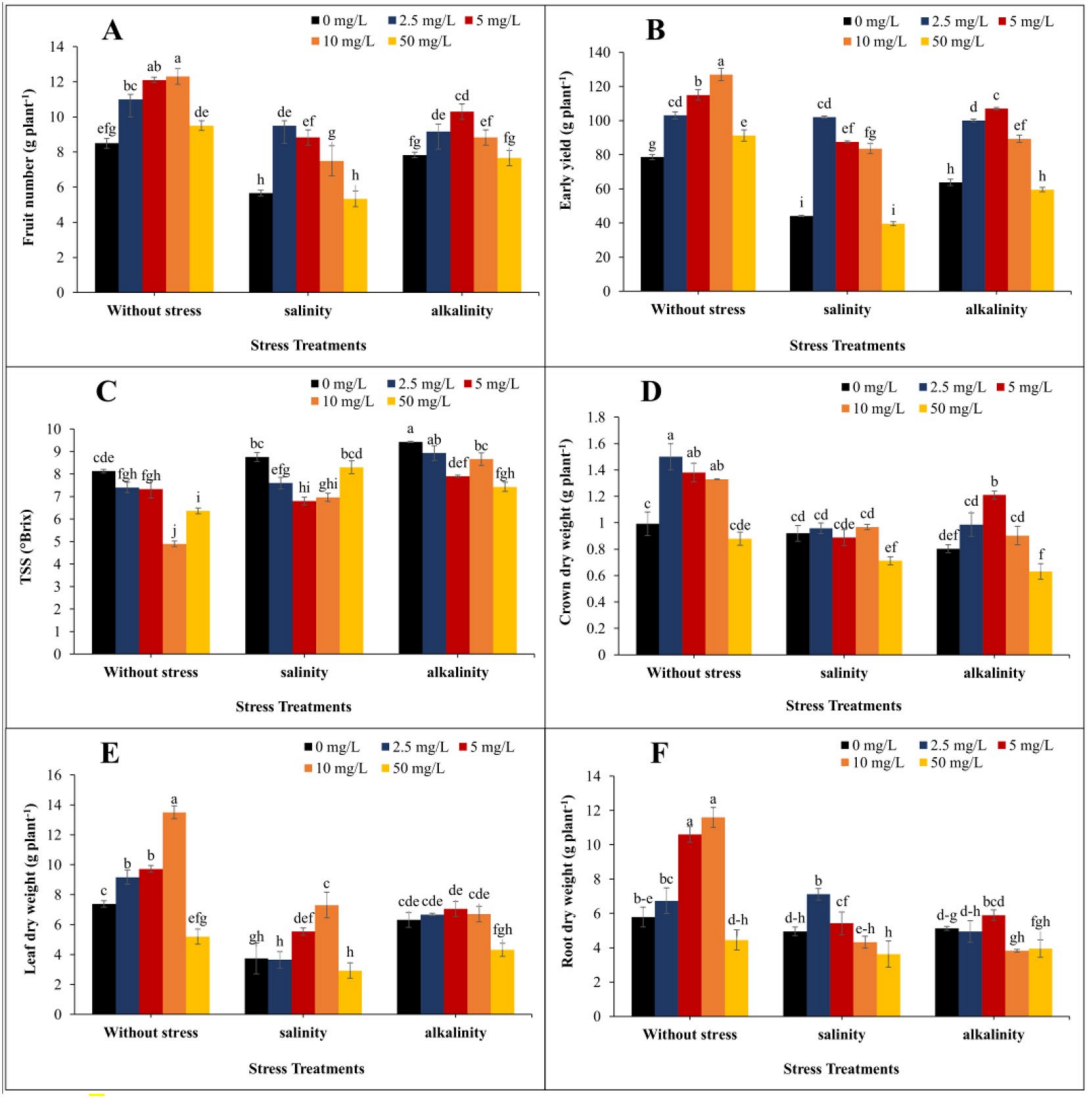
Changes in (**A**) fruit number; (**B**) early yield; (**C**) fruit TSS; (**D**) crown dry weight; (**E**) leaf dry weight; (**F**) root dry weight under different GO concentrations and three stress levels in strawberries cv. Sabrina. Means followed by the same letter for a parameter, are not significantly different according to the LSD (p ≤ 0.05). Vertical bars indicate the standard errors of three replicates.

Vegetative and reproductive parameters were affected by different concentrations of GO and stress conditions. The results showed that salinity and alkalinity stress conditions reduced the number of fruits and early yield. In without stress conditions, increasing the concentration of GO up to 10 mg/L increased the number of fruits and early yield. In salinity and alkalinity stress conditions, the highest number of fruits compared to the without stress was observed in concentrations of 2.5 mg/L (40.42%) and 5 mg/L (26.5%), respectively. The highest amount of early yield was recorded in salinity stress at a concentration of 2.5 mg/L of GO with a 131.2% increase and in alkalinity stress at a concentration of 5 mg/L of GO with a 67.7% increase (Fig. 9A,B). The stress conditions increased the TSS of the fruit, and the application of GO reduced the amount of TSS of the fruit compared to the without GO treatment (Fig. 9C).

**Figure 10:**
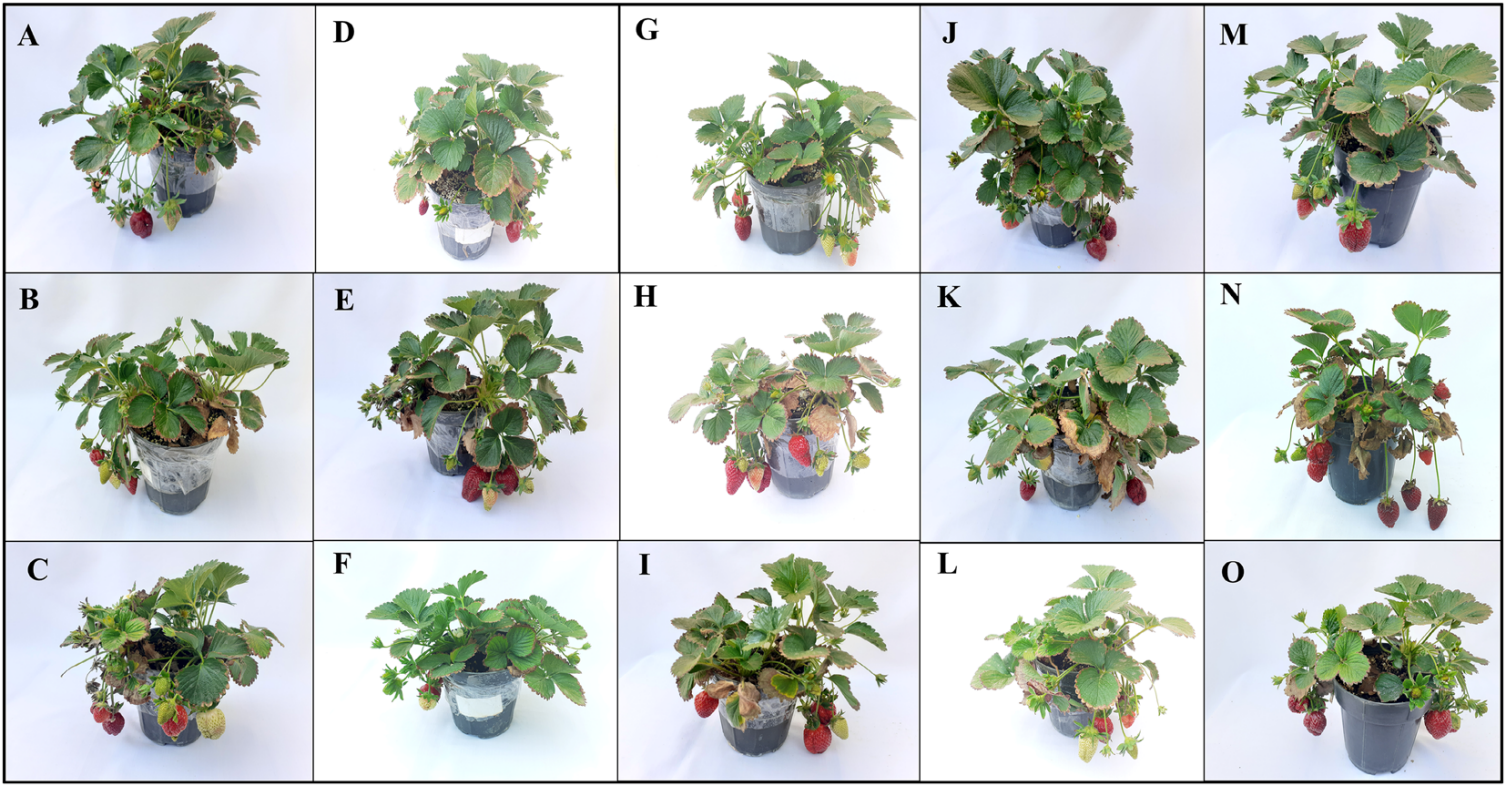
The effects of different GO concentrations under different stress levels on the morphological traits of strawberry plants. 0 mg/L GO under (**A**) without stress; (**B**) salinity stress, and (**C**) alkalinity stress. 2.5 mg/L GO under (**D**) without stress; (**E**) salinity stress, and (**F**) alkalinity stress. 5 mg/L GO under (**G**) without stress; (**H**) salinity stress, and (**I**) alkalinity stress. 10 mg/L GO under (**J**) without stress; (**K**) salinity stress, and (**L**) alkalinity stress. 50 mg/L GO under (**M**) without stress; (**N**) salinity stress, and (**O**) alkalinity stress.

Salinity and alkalinity stress significantly reduced the dry weight of the crown, leaves, and roots. Although the concentration of 2.5 mg/L of GO had the greatest effect (51%) on the increase in the dry weight of the crown in without stressed conditions. In salinity stress conditions, concentrations of Different GOs were not significantly different from each other. In alkalinity stress, the highest increase in dry weight of the crown belonged to 5 mg/L of GO with 50.6% (Fig. 9D). The highest increase in leaf dry weight compared to the treatment without GO application was in the without stress condition and concentration of 10 mg/L of GO (82.9%). In salinity stress, the treatment of 10 mg/L GO had the greatest effect (95.1%) on the increase of leaf dry weight. In alkalinity stress, different concentrations of GO were not significantly different from each other (Fig. 9E). The highest root dry weight was obtained under without stress conditions with 10 mg/L of GO. In salinity stress, the highest amount of root dry weight was obtained in the treatment of 2.5 mg/L of GO, and in alkalinity stress, the highest amount of root dry weight belonged to 5 mg/L of GO treatment (Fig. 9F). The different concentrations of GO and stress conditions had a significant effect on the vegetative and reproductive characteristics of strawberry plants (Fig. 10).

### Correlation analysis

**Figure 11:**
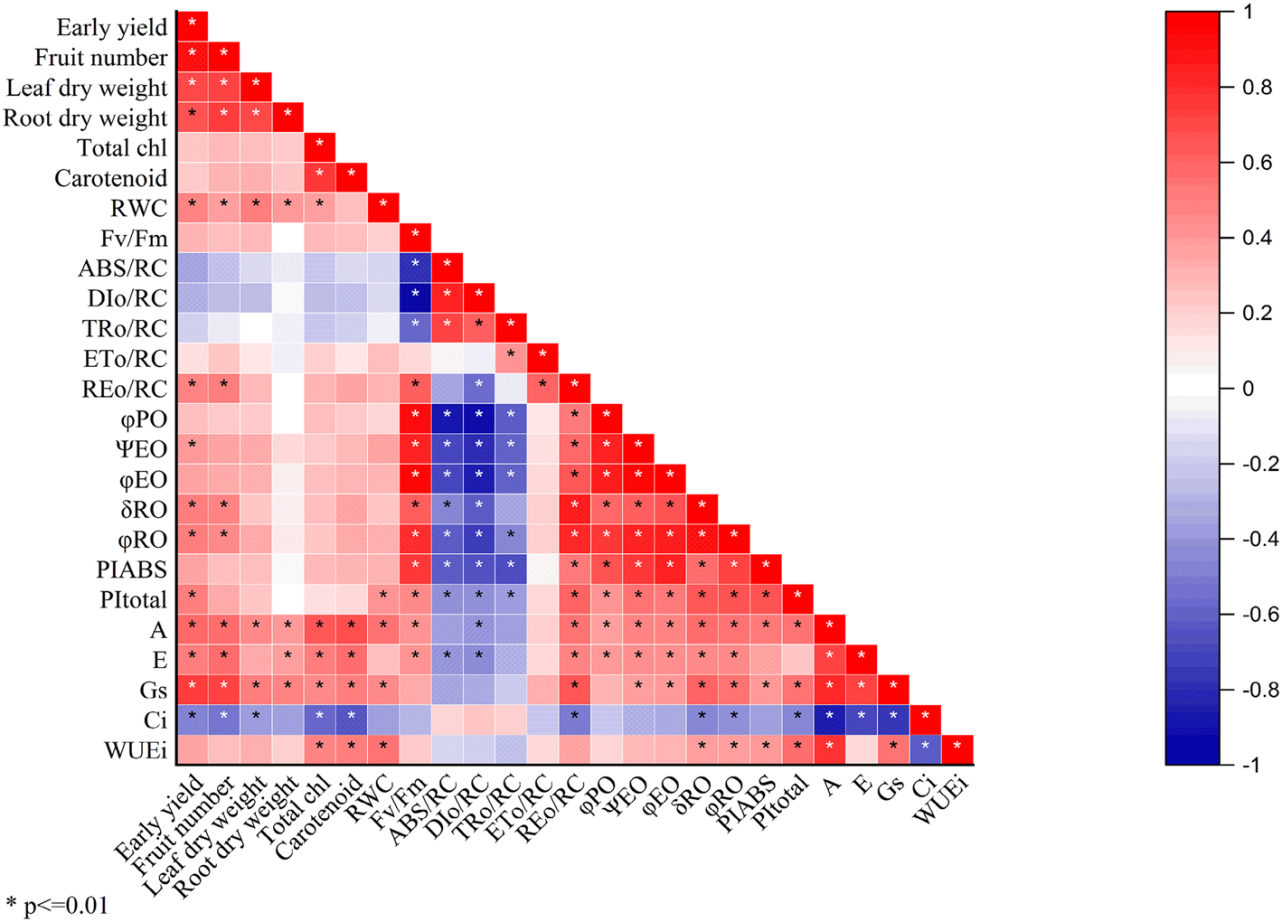
Correlation plot between vegetative, reproductive, plant gas exchange, and prompt fluorescence parameters. The size and color intensity of circles are proportional to Pearson’s correlation coefficient at p < 0.01. Red circles indicate positive correlations, while blue are negative correlations. In the correlogram scale from − 1 to + 1, Pearson’s correlation coefficient for variables is on the vertical and horizontal axis. * indicates values that are statistically different at p < 0.05.

The correlation plot (Fig. 11) shows the correlations between vegetative, reproductive, plant gas exchange, and prompt fluorescence parameters. The size and color intensity of circles are proportional to Pearson’s correlation coefficient at p < 0.01. Red circles indicate positive correlations, while blue are negative correlations. In the correlogram scale from − 1 to + 1, Pearson’s correlation coefficient for variables is on the vertical and horizontal axis. * indicates values that are statistically different at p < 0.01. The results show that the early yield positively correlated with leaf and root dry weight and fruit number. The F_v_/F_m_ parameter had a positive correlation with φ_P0_, φ_E0,_ and Ψ_E0_ and a negative correlation with DI_0_/RC. The *A* parameter was positively correlated with *WUEi*, *gs*, and *E* and negatively correlated with *C*_*i*_. The Total chlorophyll had a significant positive correlation with Total Cartenoeid and φ_P0_ had a significant negative correlation with ABS/RC and DI_0_/RC.

### Principal component analysis

**Figure 12:**
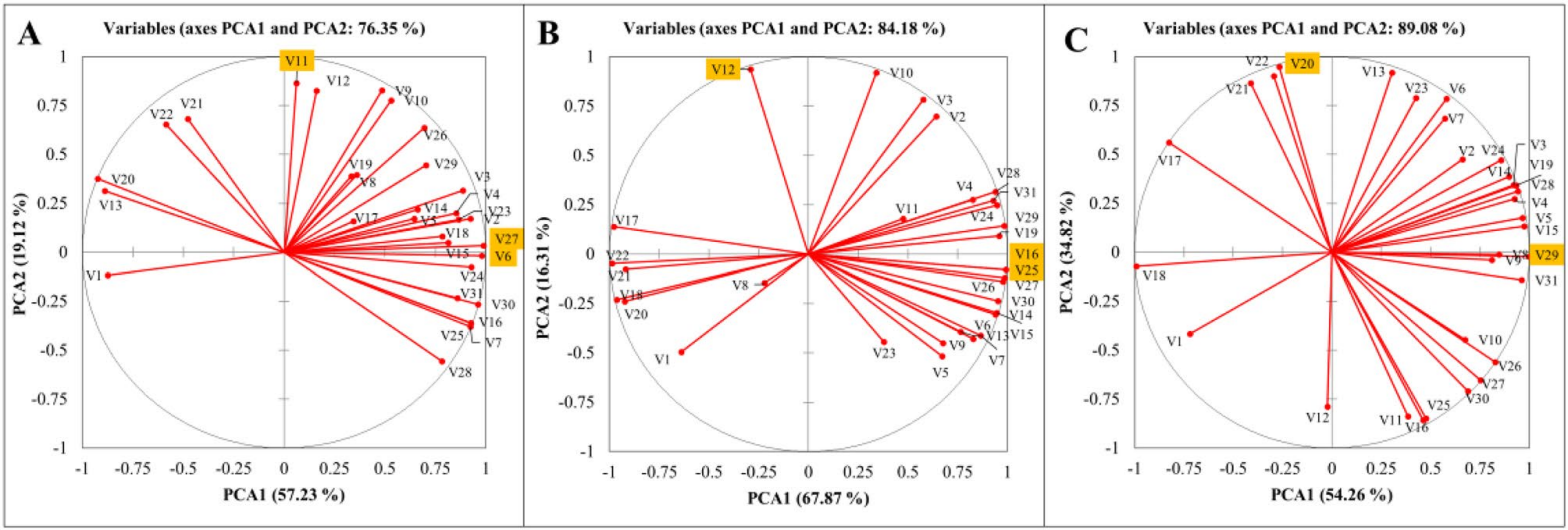
Principle component biplot of JIP test, plant gas exchange, and vegetative and reproductive parameters based on variations of 5 levels of GO concentration under (**A**) without stress; (**B**) salinity stress; (**C**) alkalinity stress. V1: *C*_*i*_; V2: *E*; V3: *g*_*s*_; V4: *A*; V5: *WUE*; V6: Total chlorophyll; V7: Total Carotenoid; V8: SPAD; V9: RWC; V10: Early yield; V11: Leaf dry weight; V12: Root dry weight; V13: F_0_; V14: F_m_; V15: F_V_; V16: F_v_/F_m_; V17: V_J_; V18: Vi; V19: Sm; V20: ABS/RC; V21: DI_0_/RC; V22: TR_0_/RC; V23: Et_0_/RC; V24: RE_0_/RC; V25: φ_P0_; V26: Ψ_E0_; V27: φ_E0_; V28: δ_R0_; V29: φ_R0_; V30: PI_(abs)_; V31: PI_(total)_.

Vector size indicates the effect of each parameter, and the direction of the vector depends on the values of PCA1 and PCA2. PCA was performed to summarize the variations of 31 parameters during five- levels of GO concentration under A: without stress; B: salinity stress; C: alkalinity stress. In the without stress treatment, the performed PCA explained 76/35% of total variations of 5 levels of GO concentration (Fig. 12A). This value was 84.18 and 89.08% for salinity and alkalinity treatments, respectively (Fig. 12B,C). Most of the variations were explained by the first component (PCA1). Regardless of the direction of the effect, parameters V6 (Total Chl) and V27 (φ_E0_) had the largest contribution to the first principal component (PCA1), and V11 (Leaf dry weight) had the highest contribution to the second principal component (PCA2) of the changes caused by the five levels of GO concentration in the without stress treatment. These parameters were V16 (F_v_/F_m_) and V25 (φ_P0_) in PCA1 and V12 (Root dry weight) in PCA2 under salinity stress treatment. Under alkalinity stress, V29 (φ_R0_) in PCA1 and V20 (ABS/RC) in PCA2 had the largest contribution to the changes caused by the five levels of GO concentration (Fig. 12).

## Discussion

The effect of salinity and alkalinity stress on the growth and photosynthesis of different plant species has been investigated^22^. This experiment focuses on investigating the effect of different concentrations of GO on increasing the tolerance of strawberry plants to salinity and alkalinity stress. Salinity and alkalinity stress have a negative effect on leaf and root dry growth, early yield, chlorophyll, and photosynthesis. High salinity increases the production of ROS, such as hydrogen peroxide (H_2_O_2_) in plants, which causes the production of hydroxyl radicals (OH) through the Fenton reaction. These radicals cause severe cell damage^23^. Treatment with 400 and 800 mg/L of GO increased the activity of catalase and ascorbate peroxidase enzymes in bean (*Vicia faba* L.) seedlings, which prevents the increase of enzyme activity from excessive production of H_2_O_2_ in the plant^24^.

The response of plants to nanomaterials depends on the properties of nanomaterials^25^. GO nanoparticles have a special structure and can easily penetrate the plant cell wall and affect the physiological and genetic processes of plants^6^. GO nanomaterials reduce oxidative stress in plants by reducing ROS under salt stress conditions^26^. The application of GO increased chlorophyll, carotenoids, SPAD, RWC, and photosynthetic efficiency, which led to increased vegetative and reproductive growth and plant dry weight. High concentrations of GO application can have a negative role on plants, and it has been shown that doses higher than 50 mg/L of GO suppressed root growth in rice plants^27^.

Salinity stress with the increase of toxic ions has led to the decomposition and reduction of photosynthetic pigments in plants^28,29^, which can be due to the damage of ROS induced oxidative stress caused by salt stress^28^. Increasing the chlorophyll biosynthesis can be considered as one of the reasons for increasing chlorophyll content by GO treatments in salinity stress conditions^30^. It is also related to the effect of GO on light absorption due to the ability of carbon-based nanoparticles to penetrate chloroplast membranes and increase the number and size of chloroplasts^12^. Foliar application of GO increases the quantum yield of PSII and PI^13^. The use of GO increases PI, Sm, N, RE_0_/Rc, and performance index significantly, which shows that GO nano-materials affect the electron transport chain and the energy pathways affect and facilitate the electron transfer from the donor side to the final acceptor in PSII^31^. GO have a positive role in photosynthesis, cell membrane stability, soluble sugar content, and expression of aquaporins under salt stress conditions by reducing sodium absorption^32^.

Although there are conflicting reports regarding the beneficial^33^ and harmful^5,34^ effects of GO on plants from different perspectives, such as biochemistry, plant physiology, molecular, and cytology, it is important to investigate and analyze the effects of GO on plants. To determine the positive effects of GO on plant growth, the appropriate concentration of GO should be determined, and the size of the GO particles used should be considered. This evaluation provides a comprehensive understanding of the effects of GO on plant growth and development for its effective use. Different concentrations of GO have different effects on plants in different conditions. The use of GO in soil up to a concentration of 50 mg/L increased vegetative growth, soluble sugar, chlorophyll content, Fv/Fm, and Y(II) in Aloe vera plants^35^. These results show that although the mechanism is not yet clear, the effects of GO are very important for the growth and adaptation of plants to stress in agriculture. By increasing the surface of roots^36^ and leaves^33^, GO provides more water and mineral nutrients for plant growth^37^, and by increasing photosynthetic pigments^8^ and improving the performance of the photosynthetic apparatus, it leads to an increase in biomass. The positive impact of GO can be attributed to its exceptional physicochemical properties, such as high electronic conductivity, high surface area, and high mechanical strength of nanoparticles^38^. These properties allow GO to act as delivery systems and imaging agents for plant cells and tissues^39^, which can improve plant growth. Our results showed the positive effect of GO on increasing photosynthetic pigments and L and K bands and reducing F_0_, but different concentrations of GO had different effects in different conditions.

Our results (Fig. 12) showed that the parameters of quantum performance and total chlorophyll contributed the most to the total changes of 5 levels of GO. GO plays a positive role in the transfer of electrons to the final receptors in PSI, and the performance of the photosynthetic apparatus increases with GO^13^. GO foliar treatments improve shoot growth, plant height, and chlorophyll under salt stress by increasing the activity of antioxidant enzymes and osmolytes and balancing minerals^15^. The planned application of nanocarbon, such as GO, could be a promising alternative to deal with the adverse effects of salinity on plant growth and development.

## Conclusions

The analysis of the photosynthetic apparatus and vegetative and reproductive characteristics showed that strawberry plants react to the application of GO. The use of GO at concentrations of 5 and 10 mg/L had a positive and significant effect on plants compared to other concentrations. By affecting the photosynthetic apparatus and the electron transport chain, GO improved the growth and development of plants under stress conditions and affected many aspects of the morphology and physiology of strawberry plants. By investigating plant gas exchange parameters and chlorophyll fluorescence as biological indicators, the effects of GO can be better understood. Although applying GO in stress conditions can be promising, the concentrations used should be investigated. Our results show that GO can play a promising role for use in agriculture as a carbon nanomaterial.

## Supplementary Information

Supplementary Table S1.

## Data Availability

Data available within the article and its Supplementary Materials.
